# Molecular Detection of Tick-Borne Bacterial Pathogens in Patients With Undifferentiated Febrile Illness in India Using a Flow Chip Assay

**DOI:** 10.7759/cureus.75781

**Published:** 2024-12-16

**Authors:** Chandan K Thakur, E. V. Vinayaraj, Rama Chaudhry

**Affiliations:** 1 Department of Clinical Microbiology, Karnali Academy of Health Sciences, Jumla, NPL; 2 Department of Microbiology, All India Institute of Medical Sciences, New Delhi, New Delhi, IND

**Keywords:** febrile illness, hybrispot, rickettsia, tick-borne bacterial flow chip, tick-borne disease, vector, zoonosis

## Abstract

Background: Tick-borne diseases (TBDs) play a crucial role in human morbidity and mortality, as ticks are highly effective in spreading diseases by transmitting harmful pathogens to humans and animals. The last few decades have seen an increase in the number of recognized tick-borne pathogens and the incidence of TBD worldwide. Several of these diseases are ubiquitous in India. However, in India, there is limited information on the molecular detection of tick-borne pathogens in patients with undifferentiated febrile illness. The study aims to investigate tick-borne pathogens among undifferentiated febrile patients in India using a multiplex polymerase chain reaction (PCR)-based assay.

Methods: A total of 120 blood samples were collected from patients with undifferentiated febrile illnesses of all ages. The samples were tested for a panel of (seven) tick-borne pathogens (*Anaplasma, Ehrlichia, Borrelia, Bartonella, Coxiella, Rickettsia, *and* Francisella*) using a multiplex PCR tick-borne bacterial flow chip (TBFC) assay through a semi-automated HybriSpot platform (Vitro Master Diagnóstica, Granada, Spain).

Results: Among the 120 samples tested, one was positive for spotted fever *Rickettsia*, two were positive for typhus group *Rickettsia*, one was positive for *Borrelia*, and two showed coinfections with *Borrelia* and *Anaplasma*.

Conclusion: Our knowledge of TBD is steadily advancing with the discovery of novel pathogens and the development of cutting-edge diagnostic tools. Although traditional diagnostic methods like serology and microscopy will remain essential for the diagnosis of TBD, the implementation of advanced molecular diagnostics such as TBFC will enhance our understanding of these diseases by enabling the identification of emerging pathogens and offering more precise and timely diagnoses.

## Introduction

Ticks are distributed worldwide and are recognized as the second most significant arthropod vectors of human pathogens, following mosquitoes [1]. In the USA, ticks are the main vector for disease, responsible for 77%-95% of the annually reported vector-borne disease cases. They can carry and transmit various bacterial, viral, and parasitic pathogens [2]. Transmission occurs when an infected tick releases pathogens into the human host through saliva while feeding on blood. The rate and likelihood of infection vary depending on the specific pathogen, with certain organisms transmitting within minutes while others may take hours to days. It has also been observed that more than one infectious pathogen can be harboured by a single tick, resulting in more than one infection in a single individual at the same time [3]. Tick-borne diseases (TBDs) mostly affect and destroy blood cells, resulting in anaemia, jaundice, haemoglobinuria, and weight loss, and tend to increase the risk for secondary infections [4]. Tick-borne infections can have a wide range of clinical presentations, varying from mild to potentially life-threatening. Common presentations include fever, headaches, rash, muscle and joint pain, nausea, vomiting, thrombocytopenia, and transaminitis, which often overlap in the early stages of the disease [5]. Since most tick-borne pathogens are challenging to culture in the lab, diagnosis mainly relies on clinical presentation, exposure history in endemic regions, examination of blood smears under a microscope, and serological tests [6]. However, direct microscopic examination necessitates a high concentration of organisms in the blood. This procedure can be time-consuming, lacking sensitivity, and subjective, often resulting in false-negative outcomes. Moreover, serologic testing has limitations, such as reduced sensitivity during the initial stages of the disease and lack of clinical specificity. Additionally, they cannot reliably distinguish between active and past infections, which can result in misdiagnoses [7]. Therefore, it is crucial to utilize rapid, sensitive, and specific multiplex polymerase chain reaction (PCR)-based tests to detect the pathogen(s) during the acute phase and guide appropriate treatment to reduce the severity of the illness. This study aimed to explore tick-borne pathogens responsible for undifferentiated febrile illness in hospital-visiting patients from India utilizing a multiplex tick-borne bacterial flow chip (TBFC) platform.

## Materials and methods

A total of 120 blood samples were selected from a cross-sectional study conducted on patients of all ages with undifferentiated febrile illness from August 2018 to August 2020 after obtaining written informed consent. No other pathogens, such as *Dengue* (NS1 antigen, Panbio™ Dengue Early ELISA, Standard Diagnostics Inc., Korea), *Leptospira* spp. (16s rRNA PCR) [8], *Salmonella* spp. (flagellin gene PCR) [9], *Plasmodium* spp. (malarial antigen, Advantage Malcard (J.Mitra, India)), or *Orientia* spp. (56kDa PCR) [10], were detected in these patients. This study was conducted at the Department of Microbiology, All India Institute of Medical Sciences, New Delhi, New Delhi, India. Ethical clearance was obtained from the Institute Ethics Committee (Ref. No.: 214/2018).

DNA extraction

The DNA was extracted from each ethylenediaminetetraacetic acid (EDTA) containing whole blood specimen (200 μL) using the QIAmp mini kit (Qiagen GmBh, Germany). DNA was eluted in 50 μL elution buffer and stored at -20°C until further use.

Detection of tick-borne pathogens

TBFC assay (Vitro Master Diagnóstica, Granada, Spain) is a DNA microarray-based test approved by the European Economic Area as a certified in vitro diagnostic device (CE IVD). It uses multiplex PCR amplification with biotin-labelled primers, followed by reverse hybridization on a membrane containing specific probes for detecting the key tick-borne pathogens linked to febrile illness. Positive results are visualized through a colourimetric immunoenzymatic reaction on a chip membrane using the HybriSpot (HS) 12 hybridization platform. The platform's external camera captures an image of the chip, which is then analysed by the HybriSoft software (HSHS 2.1.0.R10/IPL1.0.0.R0402) (Vitro Master Diagnóstica). The software identifies specific dot patterns on the membrane, each corresponding to a particular microorganism, and provides the user with the results. The assay can detect at the group/species level of seven bacteria belonging to the genus *Borrelia* spp., *Anaplasma* (*Anaplasma* spp. and *A. phagocytophilum*), *Rickettsia* (*Rickettsia* spp., typhus group (TG), and spotted fever group (SFG)), *Francisella* spp. (*F. tularensis*), *Bartonella* spp., *Coxiella* (*C. burnetii*), and *Ehrlichia* (*E. chaffeensis*, *E. ewingii *and *Candidatus Neoehrlichia mikurensis*) and also includes internal controls to monitor assay performance (Table 1). This technique enables the assay to detect a wide range of species within each genus using species-specific probes and new variants that have not yet been characterized by generic probes. The specific gene targets used for each of the seven assays are described in Table 2.

**Table 1:** Positions of probes included in the TBFC assay. B: hybridization control; CI: exogenous amplification control; BG: endogenous amplification control of beta-globin human fragment; GR: *Rickettsia* group; TG: *Rickettsia typhi* group; SFG: *Rickettsia* spotted fever group; EH: *Ehrlichia*; AN: *Anaplasma*; FR: *Francisella*; BAR: *Bartonella* 16S rRNA; BAR2: *Bartonella* gltA; BOR: *Borrelia*; COX: *Coxiella*; TBFC: tick-borne bacterial flow chip Source: Kit insert; Vitro Master Diagnóstica, Granada, Spain; https://vitro.bio/en/

B			FR				B	
B	GR				EH		BOR	
CI	TG		BAR		AN			
BG	SFG						COX	
			BAR2	B				FR
				CI				
	EH		BOR	BG	GR			BAR
	AN				TG			BAR2
	B		COX		SFG			

**Table 2:** Specific gene targets of seven tick-borne bacterial pathogens as per kit insert. Source: Vitro Master Diagnóstica, Granada, Spain; https://vitro.bio/en/

Bacteria	Gene target
*Anaplasma* spp.	16S rRNA
A. phagocytophilum	MSP2
*Ehrlichia chaffeensis, E. ewingii *and *Candidatus Neoehrlichia mikurensis*	16S rRNA
*Bartonella* spp.	16S
*Bartonella* spp.	gltA
*Borrelia* spp.	16S rRNA
Coxiella burnetii	Transposase IS1111
*Francisella* spp.	17 kDa TUL4
*Rickettsia* spp.	23S-5S
*Rickettsia* typhus group	23S-5S
*Rickettsia* spotted fever group	23S-5S

The primer sequences utilized in the TBFC assay are proprietary and, therefore, cannot be disclosed in the manuscript. The test used amplified DNA from patients’ blood with undifferentiated febrile illness. The PCR steps were carried out as per the TBFC package insert. Briefly, each PCR reaction mixture was prepared in a final volume of 40 μL in individual PCR reaction tubes (Thermo Fisher Scientific, Waltham, USA) comprising 31.2 μL of PCR mix 1/2, containing primers (forward and reverse) and specific probes of seven targeted tick-borne pathogens and primers for the amplification of two internal controls, 0.8 μL of Hot Start II DNA Polymerase, 6 μL of template DNA, and nuclease-free water (Thermo Scientific, Lithuania, EU) to achieve the final desired volume. The PCR assays were carried out using the Veriti thermal cycler system (Applied Biosystems, Foster City, USA) under the following conditions: 98°C for 5 min initial activation followed by 43 cycles of 98°C for 5-sec initial denaturation, 60°C for 5-sec annealing, 72°C for 10-sec extension, and 72°C for 1-min final extension. PCR products were combined with hybridization reagents and applied to individual chip membranes. The flow-through reverse hybridization process was semi-automatically conducted on the HS12, followed by image capture of the chip and the result was interpreted using HybriSoft software, as per the manufacturer’s instructions. To assess assay performance, the genomic DNA of *R. conorii* was used as a positive control, while nuclease-free water served as a negative control in every second run.

## Results

**Figure 1:**
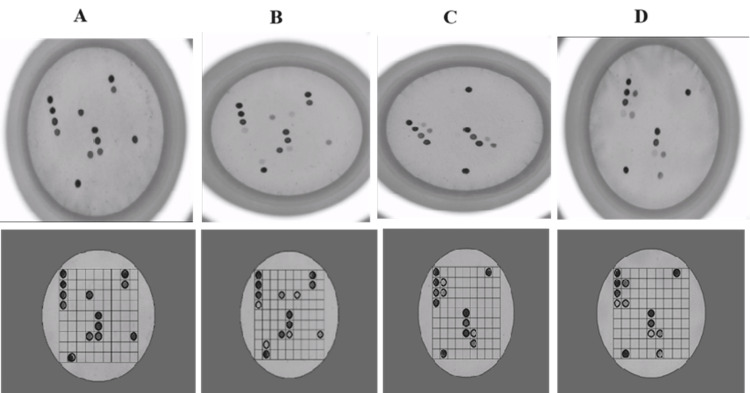
Upper images show substrate/chromogen reactions producing a dark purple precipitate (hybridization signal) where the PCR amplicon hybridized with the specific probe. Lower corresponding images display signal spots automatically captured and analyzed by the HybriSoft software. Samples tested positive for (A) *Borrelia* spp.; (B) *Anaplasma phagocytophilum* or/and *A. bovis* or/and *A. equi*, and *Borrelia* spp.; (C) *Rickettsia* typhus group; (D) *Rickettsia* spotted fever group. PCR: polymerase chain reaction

A total of six patients (5%) tested positive for TBDs. Among these, one sample tested positive for SFG *Rickettsia*, two tested positive for TG *Rickettsia*, one tested positive for *Borrelia*, and two showed coinfection with *Borrelia* and *Anaplasma*. Representative captured images and software-analyzed results are presented in Figure 1. These six samples were further tested, and the results were confirmed using specific real-time PCR assays targeting the gltA gene for pan-rickettsia, the OspA gene for *B. burgdorferi sensu lato*, and the Ank A gene of *A. phagocytophilum*, as described previously [11-13].

The mean age of patients diagnosed with TBD was 47.2±16.7 years. Males and females were equally affected (3/3). The clinical characteristics of TBD-positive and TBD-negative patients are summarized in Table 3. The most common laboratory findings of TBD-positive patients were thrombocytopenia and elevated liver enzymes. All six TBD-positive patients received appropriate antibiotic treatment, specifically doxycycline and ceftriaxone. Of these, five recovered, while one patient infected with SFG *Rickettsia* succumbed due to multiple organ dysfunction syndrome (MODS) (Table 4).

**Table 3:** General characteristics of patients positive and negative for tick-borne diseases (TBD).

Variables	TBD-negative patients (n=114)	TBD-positive patients (n=6)	P-value
Mean age	29.6±17.3	47.2±16.7	0.0165
Sex (male/female)	66/48	3/3	0.698
Rash	23 (19.8%)	2 (33.3%)	0.603
Eschar	0	0	-
Headache	31 (27.2%)	1 (16.7%)	1.000
Vomiting	29 (25.4%)	1 (16.7%)	1.000
Abdominal pain	25 (21.9%)	2 (33.3%)	0.616
Myalgia	31 (27.2%)	3 (50%)	0.349
Arthralgia	19 (16.7%)	1 (16.7%)	1.000
Jaundice	24 (21.1%)	2 (33.3%)	0.609
Splenomegaly	18 (15.9%)	1 (16.7%)	1.000
Hepatomegaly	30 (26.5%)	1 (16.7%)	1.000

**Table 4:** Clinical and laboratory findings of six patients infected with tick borne pathogens. MODS: multi-organ dysfunction syndrome

Case	Age, gender	HybriSpot results	Clinical presentation	Significant lab findings	Treatment	Outcome
Case 1	55, F	Spotted fever *Rickettsia*	Fever, macular rash, myalgia, jaundice, abdominal pain, splenomegaly, MODS	Thrombocytopenia	Ceftriaxone, piperacillin-tazobactam, doxycycline	Died
Case 2	68, M	Typhus group *Rickettsia*	Fever, myalgia, headache	Thrombocytopenia, elevated liver enzymes	Ceftriaxone, doxycycline	Recovered
Case 3	56, F	Typhus group *Rickettsia*	Fever, macular rash, myalgia, arthralgia, jaundice, abdominal pain, vomiting, hepatomegaly	Leukopenia, thrombocytopenia, elevated liver enzymes	Meropenem, doxycycline	Recovered
Case 4	27, F	*Borrelia* spp.	Facial nerve palsy and lymphocytic meningitis	Nil	Ceftriaxone, doxycycline	Recovered
Case 5	50, M	*Borrelia *spp. and *A. phagocytophilum*	Fever, bilateral facial nerve palsy, radiculoneuropathy	Thrombocytopenia and elevated liver enzymes	Ceftriaxone, doxycycline	Recovered
Case 6	27, M	*Borrelia* spp. and* A. phagocytophilum*	Fever, radiculoneuropathy	Thrombocytopenia and elevated liver enzymes	Ceftriaxone, doxycycline	Recovered

## Discussion

Tick-borne pathogens are found worldwide, including India, with ever-expanding ranges [14]. The geographic distribution of tick species is influenced by changes in micro- and macroclimate, human behaviour, travel, vector population, land use, population growth, an increase in home-based livestock settings, and various other factors. These changes have facilitated the spread of tick-borne bacterial diseases [15]. As new bacterial species are discovered, it becomes crucial to understand host transmission and closely monitor the emergence of new and existing pathogens [16].

Ticks are carriers of various pathogens, including *Rickettsia* spp., *B. burgdorferi*, *A. phagocytophilum*, and *Ehrlichia* spp., posing significant emerging vector-borne threats. Although rare, ticks can transmit *R. prowazekii i*n the TG. While the human body louse is the primary vector for *R. prowazekii,* it has also been found in *Amblyomma* and *Hyalomma* ticks in Ethiopia and *A. imitator *ticks in Mexico, though their role in disease transmission is uncertain [17]. *F. tularensis*, causing tularemia, can be transmitted through aerosols, ingestion, direct contact, and vectors like flies and ticks [18]. Although not strictly tick-associated, half of U.S. tularemia cases are linked to tick bites [19]. *Bartonella* spp., primarily flea-transmitted, have been detected in ticks, with *Rhipicephalus sanguineus* and *Ixodes ricinus *shown as competent vectors [20,21]. There has been a case of bartonellosis linked to a tick bite [22]. Q fever in humans is mainly associated with aerosol exposure, but tick bites are also a potential risk. *C. burnetii* has been found in tick-bitten patients; however, aerosol exposure cannot be ruled out in these cases [23].

Tick-borne illnesses, including rickettsioses, Lyme disease, human granulocytic anaplasmosis, tularaemia, human monocytic ehrlichiosis, and relapsing fever, are rising in India [3]. However, surveillance of tick-borne pathogens is underdeveloped despite these diseases being underreported and rapidly expanding in range. In many regions where TBD are endemic, various pathogens cocirculate, raising the risk of coinfection in both ticks and humans [24]. Individuals living in rural areas, particularly in villages near forests, face a higher risk of tick bites. The main hosts for these ticks include domestic and wild animals, small mammals, and reptiles. Adult ticks typically prefer larger mammals, while immature ticks tend to target smaller mammals. These immature ticks are key in transmitting diseases between animals and humans, as they feed on reservoir hosts that carry pathogens [25]. As social change and urbanization progress, humans, animals, and ticks increasingly share habitats, which raises the likelihood of human exposure to ticks [26,27]. The rising prevalence and transmission of TBD pose significant public health challenges. Efforts to manage these emerging diseases are hindered by the difficulty in controlling tick populations and detecting and treating infections caused by the pathogens they transmit [16]. The results of this study underscore the significant diversity of tick-borne pathogens affecting humans in India. It emphasizes the need for increased vigilance among medical professionals, public health authorities, and veterinarians to monitor and address the presence of these pathogens within the country.

This TBFC approach was previously applied in Norway and Spain to successfully identify tick-borne bacterial agents from human clinical samples [28,29]. This technology has been utilized in India at our centre for the first time to detect tick-borne bacterial infections in patients suffering from undifferentiated febrile illness. The results of this study revealed the presence of several tick-borne pathogens in India. SFG *Rickettsia* was detected in one sample, TG *Rickettsia* in two samples, *Borrelia* in one sample, and coinfection with *Borrelia* and *Anaplasma* in two samples. Several studies in India have investigated TBDs using a variety of methodologies targeting different pathogens. For example, our previous research employed serological assays, including enzyme-linked immunosorbent assay (ELISA) and immunofluorescence assay (IFA), to detect *Rickettsia* infections in patients with acute febrile illness [30]. Our findings revealed a significant TG and SFG *Rickettsia* prevalence, which aligns with previously published literature. Other studies from India have also identified *Borrelia* and *Anaplasma* as prominent pathogens in febrile patients using similar serological assays [31,32]. Consistent with these findings, our present study also identified co-infections of *Borrelia* and *Anaplasma* in two samples. While the serological methods used in previous studies were effective, they are often costly, not widely accessible, and may miss cases with low pathogen loads or those in the early stages of infection. The TBFC assay used in the current study, which employs a multiplex PCR approach to detect multiple pathogens simultaneously, is more affordable and represents a significant advancement over these conventional techniques. This study demonstrated the assay's capability to identify multiple tick-borne pathogens in a single test, which is crucial for timely and accurate diagnosis, particularly in cases of co-infection.

Previous research has also documented the presence of *C. burnetii*, *F. tularensis*, and *Bartonella* spp. associated with human infections across India [33-35]. However, in our current study, we did not detect these infections in patients with undifferentiated febrile illness. Isolating these pathogens from clinical samples is often challenging and requires specialized BSL-3 facilities. Nonetheless, using the TBFC platform in this study offers enhanced sensitivity and specificity, underscoring the importance of advanced molecular diagnostic tools in clinical settings.

The overlapping vectors, geographical regions, and transmission cycles of *A. phagocytophilum* and *B. burgdorferi*, which cause anaplasmosis and Lyme disease respectively, often lead to coinfection [36]. Coinfections are typically linked to more severe illness and ongoing complications. Hence, it is highly advantageous to use multiplex diagnostic methods to simultaneously detect these pathogens [36]. These findings provide valuable insights into the prevalence of tick-borne pathogens among patients with undifferentiated febrile illness in India. Patients with SFG and TG *Rickettsia* exhibited symptoms such as rash, myalgia, abdominal pain, and vomiting without recent travel outside their residing area. Their negative serological results indicate an acute stage of infection that standard laboratory tests ordered by clinicians would have missed. These findings underscore the importance of employing sensitive and comprehensive testing, such as multiplex molecular assays, for patients with generalized symptoms of tick-borne illnesses.

The use of multiplex PCR tick-borne flow chip assay proved to be an effective method for detecting multiple tick-borne pathogens simultaneously [29]. This advanced molecular diagnostic approach allows for detecting emerging pathogens, providing a more comprehensive understanding of TBD. Compared to conventional diagnostic methods such as serology and microscopy, the tick-borne flow chip assay offers several advantages, including improved accuracy and timely diagnosis. However, positive findings from such techniques suggest exposure to specific pathogens but do not confirm the presence of a viable tick-borne pathogen in a patient. Therefore, diagnosing TBD requires taking into account the patient’s symptoms, medical history, anamnesis, comorbidities, and molecular techniques [37].

The limitation of this study is that it included only 120 patients. While this provides valuable initial insights, a larger patient sample is necessary for a more comprehensive understanding. Expanding the study to include more patients would allow for a broader analysis and help better assess the assay's performance across diverse populations and conditions.

## Conclusions

Identifying these tick-borne pathogens in patients with undifferentiated febrile illness in this study highlights the importance of considering TBD in the differential diagnosis, especially in endemic areas like India. Early detection and diagnosis can facilitate appropriate treatment and management of these infections, reducing morbidity and mortality. Continued research and surveillance efforts are crucial to enhance our understanding of TBD and their evolving epidemiology. The detection of novel pathogens and developing new diagnostic modalities, such as the TBFC assay utilized in this study, contribute to our expanding knowledge in this field. Implementing advanced molecular diagnostics alongside traditional methods will improve our ability to identify and characterize tick-borne pathogens, ultimately leading to better prevention, control, and treatment strategies for these diseases.

## References

[1] Zhao N, Pan K, Teng Z (2023). Molecular detection reveals diverse tick-borne bacterial and protozoan pathogens in two tick species from Yingshan County of Hubei Province, China in 2021-2022. Front Microbiol.

[2] Shakir SM, Mansfield CR, Hays ED, Couturier MR, Hillyard DR (2020). Evaluation of a novel high-definition PCR multiplex assay for simultaneous detection of tick-borne pathogens in human clinical specimens. J Clin Microbiol.

[3] Negi T, Kandari LS, Arunachalam K (2021). Update on prevalence and distribution pattern of tick-borne diseases among humans in India: a review. Parasitol Res.

[4] Inci A, Yildirim A, Duzlu O, Doganay M, Aksoy S (2016). Tick-borne diseases in Turkey: a review based on one health perspective. PLoS Negl Trop Dis.

[5] Biggs HM, Behravesh CB, Bradley KK (2016). Diagnosis and management of tickborne rickettsial diseases: Rocky Mountain spotted fever and other spotted fever group rickettsioses, ehrlichioses, and anaplasmosis - United States. MMWR Recomm Rep.

[6] Pace EJ, O’Reilly M (2020). Tickborne diseases: diagnosis and management. Am Fam Physician.

[7] Buchan BW, Jobe DA, Mashock M, Gerstbrein D, Faron ML, Ledeboer NA, Callister SM (2019). Evaluation of a novel multiplex high-definition PCR assay for detection of tick-borne pathogens in whole-blood specimens. J Clin Microbiol.

[8] Mérien F, Amouriaux P, Perolat P, Baranton G, Saint Girons I (1992). Polymerase chain reaction for detection of Leptospira spp. in clinical samples. J Clin Microbiol.

[9] Chaudhry R, Laxmi BV, Nisar N, Ray K, Kumar D (1997). Standardisation of polymerase chain reaction for the detection of Salmonella typhi in typhoid fever. J Clin Pathol.

[10] Furuya Y, Yoshida Y, Katayama T (1991). Specific amplification of Rickettsia tsutsugamushi DNA from clinical specimens by polymerase chain reaction. J Clin Microbiol.

[11] Stenos J, Graves SR, Unsworth NB (2005). A highly sensitive and specific real-time PCR assay for the detection of spotted fever and typhus group Rickettsiae. Am J Trop Med Hyg.

[12] Ivacic L, Reed KD, Mitchell PD, Ghebranious N (2007). A LightCycler TaqMan assay for detection of Borrelia burgdorferi sensu lato in clinical samples. Diagn Microbiol Infect Dis.

[13] Dong T, Qu Z, Zhang L (2013). Detection of A. phagocytophilum and E. chaffeensis in patient and mouse blood and ticks by a duplex real-time PCR assay. PLoS One.

[14] Pradeep J, Anitharaj V, Sangeetha B (2024). Human rickettsial infections in India - a review. J Vector Borne Dis.

[15] Wikel SK (2018). Ticks and tick-borne infections: complex ecology, agents, and host interactions. Vet Sci.

[16] Madison-Antenucci S, Kramer LD, Gebhardt LL, Kauffman E (2020). Emerging tick-borne diseases. Clin Microbiol Rev.

[17] Bechah Y, Capo C, Mege J-L, Raoult D (2008). Epidemic typhus. Lancet Infect Dis.

[18] Troha K, Božanić Urbančič N, Korva M, Avšič-Županc T, Battelino S, Vozel D (2022). Vector-borne tularemia: a re-emerging cause of cervical lymphadenopathy. Trop Med Infect Dis.

[19] Rosenberg R, Lindsey NP, Fischer M (2018). Vital signs: trends in reported vectorborne disease cases - United States and territories, 2004-2016. MMWR Morb Mortal Wkly Rep.

[20] Wechtaisong W, Bonnet SI, Lien YY, Chuang ST, Tsai YL (2020). Transmission of Bartonella henselae within Rhipicephalus sanguineus: data on the potential vector role of the tick. PLoS Negl Trop Dis.

[21] Król N, Militzer N, Stöbe E, Nijhof AM, Pfeffer M, Kempf VA, Obiegala A (2021). Evaluating transmission paths for three different Bartonella spp. in Ixodes ricinus ticks using artificial feeding. Microorganisms.

[22] Seo JW, Kim CM, Yun NR, Kim DM, Kim SS, Choi S, Chu H (2020). Scalp eschar and neck lymphadenopathy after tick bite (SENLAT) caused by Bartonella henselae in Korea: a case report. BMC Infect Dis.

[23] Kim CW, Kim DM, Kim CM, Yun NR, Chatterjee S (2023). Coxiella burnetii infection in a patient with tick bite. J Infect Dev Ctries.

[24] Tokarz R, Tagliafierro T, Cucura DM, Rochlin I, Sameroff S, Lipkin WI (2017). Detection of Anaplasma phagocytophilum, Babesia microti, Borrelia burgdorferi, Borrelia miyamotoi, and Powassan virus in ticks by a multiplex real-time reverse transcription-PCR assay. mSphere.

[25] Ragini G, Raju HK, Krishnamoorthi R, Elango A, Muthukumaravel S, Kumar A (2023). The molecular detection of bacterial infections of public health importance in hard tick (Ixodidae) nymphs collected from the forest fringes of Western Ghats in the Goa, Karnataka and Maharashtra states of India. Microorganisms.

[26] Matos AL, Curto P, Simões I (2022). Moonlighting in Rickettsiales: expanding virulence landscape. Trop Med Infect Dis.

[27] Jin X, Liao J, Chen Q (2023). Diversity of Rickettsiales bacteria in five species of ticks collected from Jinzhai County, Anhui Province, China in 2021-2022. Front Microbiol.

[28] Quarsten H, Grankvist A, Høyvoll L (2017). Candidatus Neoehrlichia mikurensis and Borrelia burgdorferi sensu lato detected in the blood of Norwegian patients with erythema migrans. Ticks Tick Borne Dis.

[29] Jado I, Escudero R, Espigares B (2020). Rapid and highly sensitive DNA flow technology platform to detect tick-borne bacterial pathogens in clinical samples. Vector Borne Zoonotic Dis.

[30] Thakur CK, V VE, Sagar T, Das BK, Kabra SK, Wig N, Chaudhry R (2023). Serological profile of patients suspected with non-scrub typhus rickettsioses. Indian J Med Microbiol.

[31] Tilak R, Karade S, Yadav AK (2024). Lyme Borreliosis, a public health concern in India: findings of Borrelia burgdorferi serosurvey from two states. Med J Armed Forces India.

[32] Vinayaraj EV, Thakur CK, Negi P (2024). Epidemiological, clinical, and laboratory characteristics of human granulocytic anaplasmosis in North India. J Clin Microbiol.

[33] Sahu R, Rawool DB, Dhaka P (2021). Current perspectives on the occurrence of Q fever: highlighting the need for systematic surveillance for a neglected zoonotic disease in Indian subcontinent. Environ Microbiol Rep.

[34] Sholeh M, Moradkasani S, Esmaeili S (2024). Epidemiology of tularemia in the countries of the WHO Eastern Mediterranean Region (EMRO): a systematic review and meta-analysis. PLoS Negl Trop Dis.

[35] Chaudhry R, Kokkayil P, Ghosh A (2018). Bartonella henselae infection in diverse clinical conditions in a tertiary care hospital in north India. Indian J Med Res.

[36] Schlachter S, Chan K, Marras SA, Parveen N (2017). Detection and differentiation of Lyme spirochetes and other tick-borne pathogens from blood using real-time PCR with molecular beacons. Methods Mol Biol.

[37] Banović P, Piloto-Sardiñas E, Mijatović D (2023). Differential detection of tick-borne pathogens in human platelets and whole blood using microfluidic PCR. Acta Trop.

